# Size, separation, structural order, and mass density of molecules packing in water and ice

**DOI:** 10.1038/srep03005

**Published:** 2013-10-21

**Authors:** Yongli Huang, Xi Zhang, Zengsheng Ma, Wen Li, Yichun Zhou, Ji Zhou, Weitao Zheng, Chang Q. Sun

**Affiliations:** 1 Key Laboratory of Low-Dimensional Materials and Application Technologies (Ministry of Education) and Faculty of Materials, Optoelectronics and Physics, Xiangtan University, Hunan 411105, China; 2 NOVITAS, School of Electrical and Electronic Engineering, Nanyang Technological University, Singapore 639798; 3 Center for Coordination Bond and Electronic Engineering, College of Materials Science and Engineering, China Jiliang University, Hangzhou 310018, China; 4 State Key Laboratory of New Ceramics and Fine Processing, Department of Materials Science and Engineering, Tsinghua University, Beijing 100084, China; 5 School of Materials Science, Jilin University, Changchun 130012, China; 6 These authors contributed equally to this work.

## Abstract

The structural symmetry and molecular separation in water and ice remain uncertain. We present herewith a solution to unifying the density, the structure order and symmetry, the size (H-O length d_H_), and the separation (d_OO_ = d_L_ + d_H_ or the O:H length d_L_) of molecules packing in water and ice in terms of statistic mean. This solution reconciles: i) the d_L_ and the d_H_ symmetrization of the O:H-O bond in compressed ice, ii) the d_OO_ relaxation of cooling water and ice and, iii) the d_OO_ expansion of a dimer and between molecules at water surface. With any one of the d_OO_, the density ρ(g·cm^−3^), the d_L_, and the d_H_, as a known input, one can resolve the rest quantities using this solution that is probing conditions or methods independent. We clarified that: i) liquid water prefers *statistically* the mono-phase of tetrahedrally-coordinated structure with fluctuation, ii) the low-density phase (supersolid phase as it is strongly polarized with even lower density) exists only in regions consisting molecules with fewer than four neighbors and, iii) repulsion between electron pairs on adjacent oxygen atoms dictates the cooperative relaxation of the segmented O:H-O bond, which is responsible for the performance of water and ice.

Water and ice has attracted much attention because of its anomalies pertaining to issues from galaxy to geology, astrophysics, biology, climate, and to our daily lives^1^,^2^,^3^,^4^,^5^,^6^,^7^. However, the structure order, the geometric symmetry, the size and the separation between molecules packing in water and ice (H_2_O) and their correlation remain yet highly disputed, independent issues despite decades-long intensive investigation. For instances, the separation between adjacent oxygen atoms (d_OO_) was measured to vary from 2.70 to 3.00 Å^8^,^9^,^10^,^11^,^12^,^13^,^14^,^15^,^16^,^17^,^18^,^19^,^20^ and the molecular size (the H-O bond length d_H_) changes from 0.970 to 1.001 Å^21^. A H_2_O molecule demonstrates high *instantaneous* asymmetry with coordination numbers varying from two^22^ to four or even greater^23^. The geometric structure of the weekly-ordered H_2_O liquid was interpreted in terms of either the monomial-phase of tetrahedrally-coordinated structures with thermal fluctuation^2^,^24^,^25^,^26^ or the mixed-phase of low- and high-density fragmentation^27^,^28^,^29^. However, uncertainties in these seemingly independent issues determine jointly the density of water and ice that is macroscopically detectable but the correlation among these quantities is often ignored in consideration. This fact serves as one essential constraint for the solution to the uniqueness of structure order and molecular separation, in terms of statistic expectation, that water molecules prefer. Therefore, these structural and dimensional discrepancies can be resolved simultaneously based on the framework reported in this Letter without needing any assumption or approximation.

## Results

**Figure 1:**
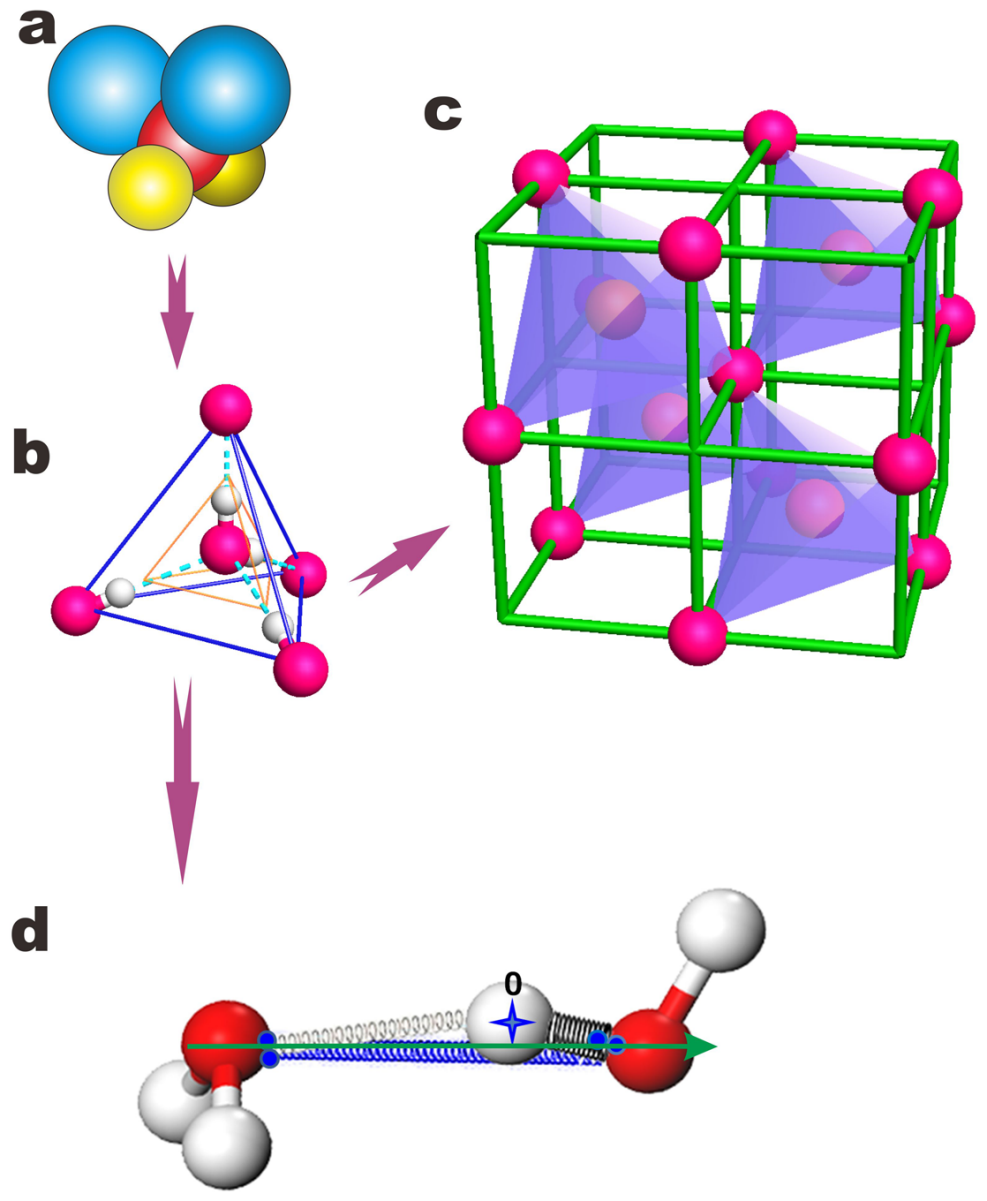
Sampling procedure for the structure of water and the segmented O:H-O bond. (a) An extension of (a) the sp^3^-hybridized oxygen (red) motif with two nonbonding electron lone pairs (blue) and two bonding electron pairs (yellow) results in (b) an ideal tetrahedron that contains two equivalent H_2_O molecules connected by four identical O:H-O bonds of different orientations. The packing of the basic building blocks (b) forms (c) a diamond structure, which ensures the tetrahedral coordination of the central oxygen atom in the coordination origin. Therefore, only four of the eight cubes in (c) are occupied by (b) and the rest four remain empty. (d) The O:H-O bond forms an asymmetric, coupled, H-bridged oscillators whose relaxation in length and energy and the associated local charge distribution determine the physical properties of water and ice^26^,^32^,^35^. Small pairing dots on oxygen represent the electron pairs.

Firstly, the sp^3^-orbital hybridization is the unique choice of oxygen upon reacting with atoms of relatively lower electronegativity, irrespective of the structural phase^30^. As shown in Figure 1a, an oxygen atom (2s^2^2p^4^) catches two electrons from neighboring atoms such as hydrogen (H) and metals and then hybridizes its *sp* orbits with tetrahedrally directional orbits^26^. In the case of H_2_O, one O forms two intramolecular H-O bonds with shared electron pairs and ~ 4.0 eV binding energy^26^ and fills up the rest two orbits with its nonbonding electron lone pairs “:” to form the intermolecular O:H non-covalent bonds of < 0.1 eV binding energy^31^. The inhomogeneous distribution of charge and energy around the central oxygen atom entitles a H_2_O molecule only C_v2_ group symmetry except for the rotation and vibration of the molecule. Therefore, an oxygen atom always tends to find four neighbors to form a stable tetrahedron but the nonequivalent bond angles (∠H-O-H < 104.5° and ∠H:O:H > 109.5°) and the repulsion between electron pairs on oxygen^26^,^32^ refrain the steady tetrahedron from being formed in the liquid phase. The strong fluctuation proceeds more like the motion of a complex pendulum surrounded by four non-bonding lone pairs, because of the O:H bond switching on and off restlessly in a period of sub-picosecond^2^,^25^,^28^,^29^. Therefore, it would be more realistic and meaningful to consider the statistic expectation of the coordination number, the structure order, and the molecular separation in all phases at question for a long time span rather than seeking for the instantaneous accuracy of a certain independent quantity by taking the snapshot at a quick flash^25^ for the highly correlated and fluctuating system.

Secondly, the packing order of H_2_O molecules follows Pauling's Ice Rule^33^ in all phases except for water under extremely high temperature and high pressure^34^. Despite thermal fluctuation in the O:H non-covalent bond lengths and the ∠O:H-O bond angles, the average separation and the size of molecules will change when the H_2_O transits from the strongly-ordered solid phase, to the weakly-ordered liquid phase, and to the disordered amorphous or vapor phase, as the Ice Rule retains. An extension of the Ice Rule results in an ideal tetrahedron, shown in Figure 1b, with higher C_3_ group symmetry. This tetrahedron containing two equivalent H_2_O molecules and four identical O:H-O bonds at different orientations forms the basic block building up the bulk water and ice despite fluctuations.

Thirdly, as illustrated in Figure 1c, four of the eight cubes are occupied by the basic 2H_2_O block tetrahedrally and the rest four cubes are empty, which means that each cube of a^3^ volume accommodates only one H_2_O molecule on average. With the known mass of a H_2_O molecule consisting 8 neutrons, 10 protons, and 10 electrons, M = (10 × 1.672621 + 8 × 1.674927 + 10 × 0.000911) × 10^−27^ kg and the known density ρ = M/a^3^ = 1 (gcm^−3^) at 4°C under the atmospheric pressure, this structural order defines immediately and unambiguously the density-dependent molecular separation, d_OO_, and the next-nearest neighboring distance √2a (unit in Å), 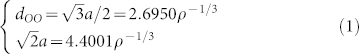
Finally, the O:H-O bond, in Figure 1d, consists of the longer-and-softer part of the O:H van der Waals bond (d_L_) and the shorter-and-stiffer part of the H-O polar-covalent bond (d_H_) rather than either of them alone. The O:H-O bond approximates a pair of asymmetric and H-bridged oscillators coupled by Coulomb-repulsion, whose relaxation in length and energy and the associated local charge distribution determine the anomalies of water ice under various stimuli such as compression^32^, coordination number reduction^26^, and cooling^1^,^7^,^35^. Under excitation, oxygen atoms dislocate along the O:H-O bond in the same direction but by different amounts with H atom as the coordination origin. The O:H-O interaction in Figure 1d holds statistically true in any phase including amorphous despite the strong fluctuations whose extent is subject to the thermal conditions due to the switching on and off the O:H interactions.

**Figure 2:**
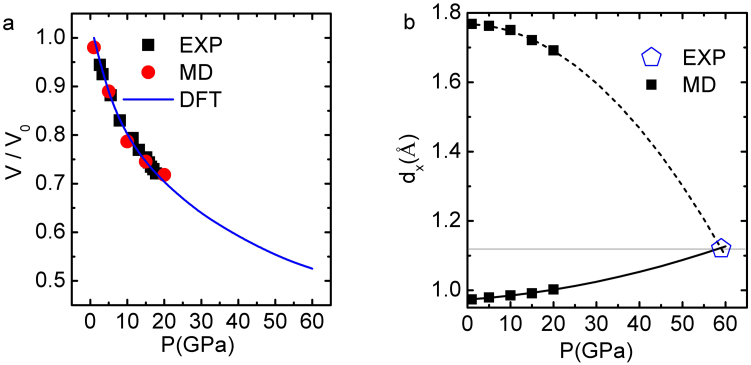
(a) MD calculation reproduction of the V(P) profile of ice^36^ with derivatives of the O:H and H–O lengths meeting at d_H_ = d_L_ = 1.12 Å under 58.6 GPa compression, which agrees with the measurements of d_H_ = d_L_ = 1.12 Å at 59 GPa^38^,^39^.

A molecular dynamics (MD) computation has enabled us to decompose the measured volume-pressure V(P) profile of compressed ice^36^,^37^ into the d_H_(P) and the d_L_(P) cooperative curves^32^, see Figure 2. The d_x_(P) curves meet at d_L_ = d_H_ = 1.12 Å under ~ 59 GPa pressure of ice, which is exactly the measured proton symmetrization of hydrogen bond in ice^38^,^39^.

This coincidence indicates that the MD derived d_x_(P) relation represents the true cooperativity of the d_L_ and the d_H_ bond relaxation. Plotting the d_L_(P) against the d_H_(P) yields immediately the (projection along the O—O) length cooperativity that is free from probing conditions or probing methods, 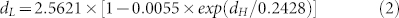
The *d*_x_ (x = L and H) value approaches the true bond length with ~ 1.5% deviation (1-cos(10°) = 0.015) as the O:H-O angle remains 160° in liquid and greater in solid^35^. Combining eqs (1) and (2), one is able to scale the size d_H_ and the separation d_OO_ of H_2_O molecules with the given packing order in Figure 1c and the measured density under various conditions. If the d_OO_ or the d_H_ matches those of direct measurement, the structure order in Figure 1c and eqs (1) and (2) are justified true and unique.

**Figure 3:**
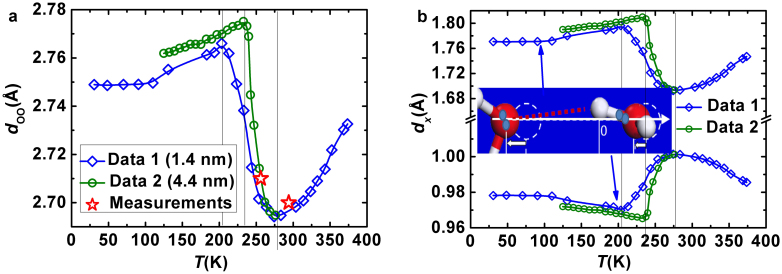
(a) The d_OO_ ~ ρ(T) profiles (eq 1) of water droplets of different sizes (1.4 and 4.4 nm)^40^,^41^ match the d_OO_ values measured at 25°C and −16.8°C (Figure S1 and S2 of Supporting Information)^10^. (b) The d_H_ and the d_L_ (eq 2) agrees with results of MD calculations^35^. Inset (b) illustrates the cooperative relaxation of the segmented O:H-O bond. One part becomes longer; the other part will be shorter by different amounts due to the inter-electron pair repulsion.

Using eq 1, one can convert, as shown Figure 3a for instance, the measured density ρ(T) profiles of water droplets of different sizes (1.4 and 4.4 nm)^40^,^41^ as input into the d_OO_ as an output for water at different temperatures. The density transition points change with water droplet size. For droplet of 1.4 nm, the transition is at 205 K, it is at 242 K for 4.4 nm droplet and 258 K for the bulk water^35^. The droplet size discriminated density transition arises from the specific heat disparity of the O:H- and the H-O within the O:H-O bond. As the droplet size is reduced, the H-O bond becomes shorter and stiffer yet the O:H bond the otherwise^26^, which shifts the cross points of the two specific heat to temperatures outwardly away from that of the bulk (refer to Ref. 35). The d_OO_ in a water droplet expands additionally in the skin region^42^ but one can only measure its average^26^. The d_OO_ values of 2.70 Å measured at 25°C and 2.71 Å at −16.8°C^10^ match exactly the conversion of 2.6950 Å that is a projection along the O—O at 4°C. This consistency justifies sufficiently that both eq 1 and the packing order in Figure 1c describe the true situations in both water and ice. Furthermore, the data reported in Ref. 10 is essentially accurate and correct.

## Discussion

The non-covalent bond length d_L_, molecular size d_H_, molecular separation d_OO_, and the mass density ρ can be obtained by solving the equation with any one of these parameters as a known input, 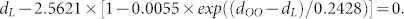


Figure 3b shows the decomposition of the d_OO_ into the d_x_ of water and ice at cooling^40^,^41^. The d_x_(T) profiles follow the rules of O:H-O bond relaxation^26^,^32^,^35^: i) both oxygen atoms dislocate in the same direction (see inset) along the O:H-O bond by different amounts with respect to the H atom; ii) the longer-and-softer O:H part always relaxes more than the shorter-and-stiffer H-O part does. The cooperativity of the d_x_ relaxation confirms further that^35^: i) cooling contraction happens only to the O:H bond in the solid (T < 205 K (Data 1) or 241 K (Data 2)) and in the liquid phase (T > 277 K), which lengthens the H-O bond slightly by inter-electron-pair repulsion, resulting volume contraction; ii) in the freezing transition phase, the process of length relaxation reverses, leading to the O—O length gain and volume expansion at freezing.

**Figure 4:**
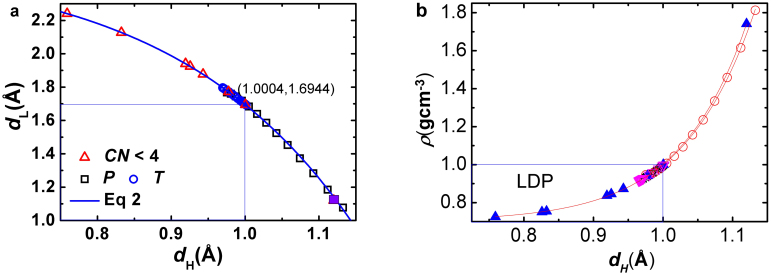
Accordance of (a) molecular size d_H_, molecular separation d_L_(or d_OO_ = d_H_ + d_L_), (b) mass density ρ, and packing order (see Figure 1c) of H_2_O molecules in the situations: (i) ice under compression (d_H_ > 1.00 Å)^36^, (ii) water ice at cooling (0.96 < d_H_ < 1.00 Å)^40^,^41^, and (iii) liquid surface and dimer (d_H_ < 1.00 Å)^8^,^9^,^10^,^14^,^15^,^16^,^17^,^18^. The derived d_H_ = 1.0004 Å at ρ = 1 is within the measurements ranging from 0.970 to 1.001 Å^21^. The d_H_ shorter than 0.96 Å corresponds to the supersolid phase in regions of molecules having fewer than four coordination neighbors (CN)^19^,^20^,^26^. In such regions, a H_2_O molecule shrinks in size and expands in separation because of inter electron-pair repulsion^26^.

Figure 4 shows the solution consistency to the measured molecular size d_H_, molecular separation d_L_ (or d_OO_), mass density ρ, and structural order of: i) compressed ice^36^, ii) cooling water and ice^40^,^41^, and, iii) water surface and dimer^10^,^19^. The d_H_ of 1.0004 Å at unity density is within the measured values ranging from 0.970 to 1.001 Å^21^. The d_OO_ values greater than the ideal value of 2.6950 Å at ρ = 1 (g·cm^−3^) correspond to the supersolid phase (low-density, LDP) that exists indeed^27^,^28^,^29^ but only presents in the skins of water ice composed of molecules with fewer than four neighbors (Figure 4b)^26^.

Wilson et al^19^ have discovered that the surface d_OO_ expands by 5.9% from 2.801 to 2.965 Å at room temperature. If one considers the shortest distance of 2.70 Å^10^ and the longest 2.965 Å^19^ of measurements, the surface d_OO_ expands by up to 10%. Furthermore, the volume of water molecules confined in 5.1 and 2.8 nm TiO_2_ pores increase by 4 and 7.5%, respectively, with respect to that in the bulk^43^. With a 5–10 Å thick air gap existing in between molecules and the hydrophobic surface^44^, water molecules at the interface exhibit skin vibration attributes^45^ of 3400 cm^−1^ compared to that of 3200 cm^−1^ for the bulk water. The separation d_OO_ = 2.980 Å for a dimer is even greater.

In these supersolid regions, molecular under-coordination shortens the d_H_ and lengthens the d_L_, resulting in d_OO_ expansion and polarization because of the inter electron-pair repulsion^26^. The least density of ice is 0.92, which corresponds to d_OO_ = 2.695(0.92)^−1/3^ = 2.7710 Å. However, the density of the supersolid phase is ρ = (2.695/2.965)^3^ = 0.7509 g·cm^−3^, which is far lower than the least density of the bulk ice or the maximal density of water (0.75/0.92/1.0), according to eq 1. Considering the limitation of penetration depth in the optical reflection measurements of water and ice, all the reported data for the skin are reasonably correct.

The molecular separation d_OO_ = d_L_ + d_H_ grows and molecular size d_H_ shrinks simultaneously at the skins because of the molecular under-coordination^26^. The H-O bond contraction follows Goldschmidt-Pauling's rule of “atomic coordination number-radius” correlation^46^,^47^; the d_OO_ expansion results from the Coulomb repulsion between electron pairs on adjacent oxygen atoms^26^,^32^. The skin region, consisting molecules with fewer than four neighbors, forms such an amazing supersolid phase that possesses the attributes of low-density^19^, high elasticity^48^, polarized^49^,^50^, dielectric instability^51^, thermally stable^52^ and hydrophobic^53^,^54^ with densely entrapped bonding electrons^55^,^56^,^57^,^58^. The timescale for hydrogen-bond switching dynamics at the surface is about three times slower than that in the bulk^59^ because of the strong polarization and high viscosity.

The findings apply to any situations including solid-liquid (water-ice) interface skin as only mass and volume are involved. At the water-hydrophobic surface of different materials, this findings are only valid to the water skin that forms the low-density supersolid state of polarized, depleted, elastic, and thermally stable^26^. An air gap of 0.5 ~ 1.0 nm thick presents between the superhydrophobic substrate and water^44^.

The straightforward yet simple solution presented herewith has thus resolved the seemingly independent geometry and dimension uncertainties of water and ice. We may conclude:

- One should focus on the statistic mean of all the factors and their cooperativity involved rather than the instantaneous accuracy of the individual parameter once at a point of time for the strongly fluctuated water system.
- The size, separation, structural order, and mass density of molecules packing in water and ice are correlated, which is independent of the structural phases of water and ice or the probing conditions.
- Constrained by the Ice Rue, the d_H_ and d_L_ cooperativity, the solution has reconciled measurements of hydrogen-bond length symmetrization of ice under compression, d_OO_ relaxation of water and ice at cooling, and d_OO_ expansion of a water dimer and molecules at water surface.
- With any one of the molecular separation, mass density, O:H bond length, and H-O distance as a known input, one can determine using this solution unambiguously the rest three parameters and their change with external conditions such as pressure, temperature, and coordination environment.
- The tetrahedrally-coordinated structure could be the unique choice of water and ice despite fluctuations in the d_L_ and the ∠O:H-O angle due to the non-equivalent ∠H:O:H and ∠H-O-H bond angles and the inter-electron-pair repulsion.
- The supersolid (low-density) phase indeed exists but only in regions consisting water molecules with fewer than four neighbors. The supersolidity phase forms because of the Goldschmidt-Pauling's rule of H-O bond contraction due to molecular under-coordination and the inter-electron-pair repulsion pertaining to the O:H-O bond.

## Methods

The MD calculations were performed using Forcite's package with *ab initio* optimized forcefield Compass27^60^. The Compass27 has been widely used in dealing with the electronic structures and the hydrogen bond network of water and amorphous ices^61^ as well as water chains in hydrophobic crystal channels^62^.

## Author Contributions

X.Z. and Y.H. contribute equally in computations. Z.M. and W.L.initiated the topic of research and prepared figures. Y.Z., J.Z. and WZ. involved explanations. C.S. wrote the manuscript. All authors reviewed the manuscript.

## Supplementary Material

Supplementary Information Supporting information
