# Supplementary material for: Size, separation, structural order, and mass density of molecules packing in water and ice
Source: Sci Rep. 2013 Oct 21;3:3005. doi: 10.1038/srep03005 (PMC3801132; doi:10.1038/srep03005)
Supplement: Supplementary Information — Supporting information [file srep03005-s1.doc]

**Supporting Information**

# **Size, separation, structural order, and mass density of molecules packing in water and ice**

CQ Sun, Yongli Huang, Xi Zhang, Zengsheng Ma, Wen Li, Yichun Zhou, Ji Zhou and Weitao Zheng

***E-mail:*** [*Ecqsun@ntu.edu.sg*](mailto:Ecqsun@ntu.edu.sg)

# Nomenclature

| Electron pairs on oxygen  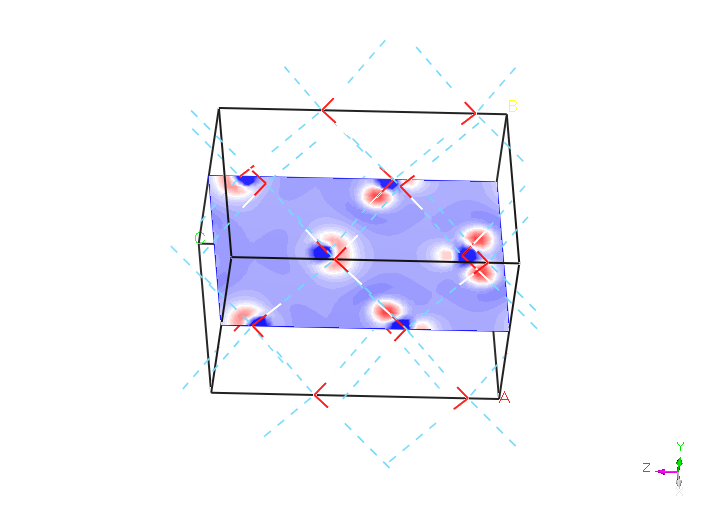 | DFT derived charge distribution in the cross-section of a unit cell of the ice-IIIV phase . Red colour represents for electron pairs and the blue for O ion cores. The electron pairs are strongly and eccentrically localized. This configuration pertains in liquid water because of the fact that the O:H and the H-O stretching phonons shift cooperatively. One segment becomes shorter and stiffer; the other will be longer and softer. |
| --- | --- |
| Short-range interactions | Interactions are within the shaded region only :   1. Intermolecular H:O van der Waals (vdW) interaction 2. Intramolecular H-O exchange interaction 3. Inter-electron-pair O---O Coulomb repulsion (C-repulsion) 4. vdW potential is estimated at E_L_ ~ 0.1 eV and the H-O bond energy at ~4.0 eV. 5. Interactions switch off out the shaded boundaries. |

**Fig S1** ρ(T) of water measured using (a) Raman and FTIR (1.4 nm) and (b) small-angle scattering of x-rays (SAXS) .

Fig S2 Fitting parameters and reliability (R-factor) of Fig 3 in main text.
